# Green product awareness effect on green purchase intentions of university students’: an emerging market’s perspective

**DOI:** 10.1186/s43093-021-00094-5

**Published:** 2021-11-28

**Authors:** Peter Ansu-Mensah

**Affiliations:** 1 Department of Marketing, Sunyani Technical University, Sunyani, Ghana; 2 Faculty of Business and Management Studies, Bono Region, Ghana

**Keywords:** Green products awareness, Green marketing, Green purchase intentions, Emerging market, Ghana

## Abstract

The indiscriminate consumption patterns worldwide have brought in its wake severe problems like pollution and global warming, and this has ultimately called for green products awareness and consumption. The main purpose of this study was to assess the effect of university students’ awareness of green products on their green purchasing intentions. The specific objectives were to identify whether awareness, price, availability, value and quality influence university students’ intention to purchase green products, and to investigate how awareness, price, availability, value and quality predict university students’ intention to purchase green products. A structural equation modeling was used to analyze data collected from an online survey of 478 students. Results show that green perceived quality has the utmost significant positive impact on university students’ green purchase intentions; however, green perceived availability had the slightest impact on university students’ intention to purchase green products. The study is the foremost to conclude that green product awareness impact on university students green purchase intentions is greatly driven by price, high value and extraordinary quality. However, availability is not a critical influencing factor when it comes to green purchase intentions of university students. The implications of study, limitations and further research are discussed.

## Introduction

The unsustainable patterns of consumption in the world today have caused severe environmental problems such as pollution, natural resources depletion, growing greenhouse gas emissions and global warming [7, 66, 69]. These aforementioned difficulties have led to the process of going “green” and eventually created attention for green products awareness and consumption [15, 56, 71]. The concept “green” is often associated with terms such as responsible consumption, ecological marketing, ecologically concerned consumer, social responsibility, natural, sustainable and environmental-friendly or pro-environmental [56, 74, 78]. Basically, these theories, responsible consumption, ecological marketing and ecologically concerned consumer, have created a platform for green consumption across the globe [27, 34, 35].

Green products have become the most reliable resolution for environmental sustainability in many developed countries [61]. The value of green products has a significant impact on the growth and development of individuals as well as the environment [25], and this is part of the reasons that the sustainable development goals (SDGs) were launched in 2015 [69]. The goal 12 of the SDG**s** stipulates “responsible consumption and production patterns” by 2030 [69]*.*

Similarly, there is a remarkable rise in environmental awareness. This surge in environmental awareness has equally increased the pressure on consumers to consider the environmental consequences of their activities in the last few decades, and this is due to increase in media attention, upsurge in pressure group events, greater consciousness of environmental harms and the growth in individuals’ awareness to green products [19, 38, 52, 54]. Consequently, many consumers’ have garnered interest in the business of going green through the use of environmental-friendly products [76]. Moreover, although there is a rise in the levels of awareness of green products in developed nations, yet this green consciousness in emerging markets remains low [46, 69]. This has, therefore, stirred up public worry in emerging economies and causing businesses to embrace green marketing targets in order to keep their customers [64].

Ghana, an emerging market in sub-Saharan Africa, over the past years has not considered many proposals intended to minimize environmental impact in the process of reducing waste, provision of access to green, quality life and maximizing efficiency. As a result, it is vital for Ghanaian consumers to alter their consumption and production forms, for if they fail to change their actions, there will be irreparable destruction that will befall the environment [7]. Thus, an improved knowledge in the environmental and social effects of products which will lead to sustainable consumption solutions is of necessity. However, before the last decade, Ghanaian consumers were not familiar with green products [5, 69]. And to the author’s understanding, no research on green products awareness has been conducted among students in an emerging market such as Ghana. The unawareness of green products withdraws the opportunity to embrace quality life as well as reduction of cost and inefficiency [38, 69].

Against this background, the aim of this study is to assess the effect of university students’ awareness of green products on their green purchase intentions. The specific objectives of this paper are as follows: 1) to identify whether awareness, price, availability, value and quality influence university students’ intention to purchase green products and 2) to find out how awareness, price, availability, value and quality predict university students’ intention to purchase green products. Consequently, the following research questions (RQs) will seek to be answered:

- RQ1: Does awareness, price, availability, value and quality influence university students’ intention to purchase green products?
- RQ2: How do awareness, price, availability, value and quality predict students’ intention to purchase green products?

This study is significant because, first, not much study on green products awareness has been conducted in emerging countries, especially in a sub-Saharan African (SSA) country. Second, to the best of the author’s knowledge, no study on green products awareness has been carried out among university students in an emerging economy such as Ghana and SSA. Third, through this study, policies could be formulated which could eventually aid in achieving tremendous green consumer behavior standard of living in order to protect the earth’s disappearing resources. Besides, this study’s findings will serve as evidence of practices that university students’ adopt in their bid to protect the environment and prevent its degradation. Additionally, this research can influence and enhance marketing and place emphasis on the exact factors, thereby helping to develop a more optimistic approach toward green purchase intentions. Similarly, an enquiry into the impact of green product awareness on consumers’ green purchase intention could offer valuable evidence to advertising agencies regarding advertising materials along with channels to be used to propagate the message of green product consumption. Moreover, the awareness of the influencing factors will certainly allow students to uphold environmental sustainability through an appeal to consumers in a more appropriate/precise manner. This can result in improved usage of environmentally friendly products that uses fewer resources and causes less harm to the environment.

## Literature review and Hypotheses development

### Green marketing

Green marketing has become very important in contemporary marketplace. Green marketing comprises all-inclusive marketing activities of packaging, product modification and manufacturing processes that are done in an environmentally harmless manner while meeting the needs of customers’ [21]. Pagliacci et al. [56] also explained green marketing as a process of bringing green products to the market devoid of harming the environment. In short, green marketing deals with a situation whereby products are marketed in an environmentally friendly manner. Understandably, environmental concern and product usefulness are the main determining factors that influence consumers green purchasing intentions [33]. Even though consumers claimed to be concerned with the environment, yet this concern is not translated into actual adoption or purchasing of green products [51, 57]. This accounts for the low engagement in sustainable consumption practices by consumers [76]. It is little wonder that Dangelico & Vocalelli [21] have opined *“environmental sustainability as the third aim beyond consumers’ satisfaction and company profitability.”* The major challenges of green marketing are the lack of public consensus on what encompasses green [62], the need to have a standard measure to tell if a product is organic or not, and requiring patience because it is a new concept [45]. Ensuring continued growth with profits and saving money are some of the benefits of green marketing [45].

### Green consumerism

The concept of the “green consumer” has become the pivot around which marketing strategies relating to the environment have been concentrated by marketing professionals and scholars [3]. Green consumers (GCs) are those consumers who make the intense effort to avoid purchasing potentially hazardous products [34]. GCs can also be referred to as those who avoid any product which causes harm or damage to any living organism and destroys the environment during manufacturing or consumption [75]. Again, GCs are considered as those consumers who are conscious and loyal to the environment [70] and knowledgeable in environmental issues [47]. GCs are those inclined with perceived behavioral control and green advertisement [39]. Although the prices of green products may be quite higher than the traditional products, GCs still patronize them because of the potential long-term gains [63]. Again, GCs consider the effect of their consumption on other people and this distinguishes them from ordinary consumers [36, 48, 56]. Moreover, the presence of GCs indicates that there should be green marketing [56].

### Green products awareness

Green products (GPs) are commodities which normally bear characteristics such as energy efficient, recyclable, low emitting, healthy products and the likes [12]. GPs are normally produced through natural friendly processes in a more durable and toxic-free manner [22]. GPs are the environmentally friendly products which production processes does not exert much influence on the environment [31, 56]. In this study, green product is explained as a term that can be used to define a product which does not eat up resources or degrades the environment and has the ability to ensure the safeguarding of the environment. Consumers should be conscious of the existence of a green product before purchases can be made. Thus, information on green products has influence on the consumer’s purchasing decisions. However, awareness of green products could be created through labeling, packaging and advertisement [63]. People who are aware and have used GPs agree to the fact that GPs help improve the environment [61]. Nguyen et al. [51] stated that if consumers are conscious of the performance of green products, then it will assist them in achieving individual environmental impact objectives. This indicates that awareness of green products can impact consumers’ decision-making which in turn can aid in restoring a more positive outlook in the market [53]. Again, the education on GPs would increase people’s behavioral intention to use GPs and consequently become green consumers [51]. GPs have good effects on the environment and human actions have effects on the environment. Green products consciously reduce waste and financial burdens [63]. As consumers become increasingly acquainted with GPs, they again become aware of the existence of the GPs and could possibly influence their green purchase intentions and subsequent behavior. Thus, green products awareness can have a positive relationship with purchasing intentions. Based on the above discussions, it is hereby suggested that:

**H1:** Green products awareness is positively influenced by university students green purchase intentions.

### Green perceived price

Price criterion usually serves as the main hindrance to green products purchase and that green consumers’ are only ready to pay a premium for a product if they realize that its attributes, designs and functions are beneficial to them, their families and posterity [38, 43, 46, 63]. But, according to Awuni et al. [10], perceived prices of green products do not scare green consumers because they are positive toward pro-environmental products and ready to pay premium prices for those goods. Again, prices of green products do not deter green consumers; thus, price does not play any prominent part in green purchase intentions [16]. Moreover, price fairness of green products boosts consumer’s perceived value and purchasing intentions. For instance, Chinese pay attention to environmental quality and, hence, their willingness to pay higher for green products [47]. Even though research suggests that consumers’ in emerging markets are prepared to pay higher price for green products [9], [73] found that consumers’ were unwilling to pay superior prices for green products. It could be deduced from the above discussions that green products price indeed affect consumers’ green purchase intentions [46]. In sum, perceived price is one of the major qualifiers for green products purchase [63], and green product’s price has higher bearing on consumers’ intention to purchase green product. Consequently, it is hypothesized that:

**H2:** Green perceived price is positively influenced by university students green purchase intentions.

### Green perceived availability

It is believed that awareness is created before the availability of a product. A study confirmed that the rate of awareness of green products (GPs) is higher than availability of the green products [77]. It is noted that the consumer’s decision to buy GPs is its availability [67]. Green products availability motivates consumers to purchase and thereby reduce risk to the environment [13]. Likewise, according to Yadav and Pathak [82], availability creates favorable conditions for consumers to buy GPs. Again, GPs must possess equal quality such as availability in order to catch the attention of consumers [82]. The availability of GPs makes consumers more concerned to the value presented by environmental safety [55]. It is also recorded that weakness in availability of GPs on the market does not allow consumers to consider green options [80]. Similarly, it is reported that the lack of availability of GPs on the market makes it unpopular and, therefore, no demand for such products [73]. Factors such as availability could not be excluded from those which influence consumers’ to purchase GPs. Availability is therefore important in choosing between brand name product and GPs. Obviously, no availability means no purchase [59]. All in all, the availability of green products has a greater gait on consumers’ intentions to purchase green products [82]. Therefore, it is proposed that:

**H3:** Green perceived availability is positively influenced by university students green purchase intentions.

### Green perceived value

Green perceived value (GPV) refers to a green product’s overall characteristics, benefits and performance in consumers thought processes. In short, GPV denotes a customer’s evaluation of the entire benefits that will be accrued to him or her from green products [3]. According to Patterson and Spreng [58], GPV is the comprehensive evaluation that a consumer does to acquire a clear value “*of a product/service between what is received and what is given based on a consumer’s environmental desires, keeping judgments, and needs to make a product environmentally friendly.”* In sum, GPV is the consumer’s imagination of the accumulated benefits from the usage of a green product. Of recent, GPV is very crucial and that it has a positive effect on marketing and environmental performance of a product and increases its purchasing intention [30]. Therefore, it is no wonder that businesses are highlighting the values of their products and increasing the purchasing intentions of their green products in order to maintain a lasting association with their customers [3, 72]. According to Akbar et al. [3], there is a positive correlation between GPV and green purchase intentions. And that perceived value is characteristics linked to the awareness of the products’ value, so that it could increase purchase intentions [3]. Also, consumers are expected to buy products when they recognized that the products’ values are higher [83]. Again, a valued product that is created will in turn increase consumers purchase intentions [29]. Besides, the importance of GPV to green purchase intentions cannot be overemphasized. Hence, it is posited that:

**H4:** Green perceived value is positively influenced by university students green purchase intentions.

### Green perceived quality

One of the important determining factors that impact the purchasing of green products is quality [68]. Perceived quality assesses the extent to which the quality of a product/service is perceived by the consumer [81]. Green perceived quality (GPQ) denotes how the quality or reliability of a green product is impacted by the consumer’s comprehensive decision [17, 81]. According to Zeithaml, Bitner & Gremler [84], perceived quality remains impacted through the consumer’s subjective judgment and ecological perspectives. When consumers are aware of the perceived quality of GPs, there is always the likelihood of the knowledge in ecological perception leading to an increase in their purchasing intentions [81]. In outlining the objectives of green marketing, Ottman et al. [55] revealed that first, the quality, utility and price of products should be established to please the consumer while influencing the ecosystem to some extent and, second, the products superior image should be developed. Consequently, consumers’ anticipation of excellent quality of green products is one of the important elements that impact their green purchasing intentions. Additionally, GPs certainly offer greater quality and worth which brings in its wake improved health and high standard of living [81]. What is more, the quality of GPs has a higher bearing on consumers’ purchasing intentions. As a result, perceived quality is a major qualifier for GPs purchase [63]. The results of Ritter et al. [63] suggest that the quality of GPs is positively related to green purchasing intentions. Furthermore, GPs quality far exceeds that of non-green products [26]. Consequently, the quality of GPs would be perceived positively by consumers’ if they envisage that a particular product has the attributes of being green [81]. From the above discussions, it is suggested that [69]

**H5:** Green perceived quality is positively influenced by university students green purchase intentions.

### Green purchase intention

It must be noted that consumers’ intention indeed plays a crucial part in marketing strategies. Purchasing intention refers to whatever consumers’ ponder and plan to purchase. Consumers’ behavioral intention is referred to as the possible behaviors which induce consumers commitment or decision to purchase a particular product [1]. Similarly, behavioral intention is defined as *“indications of how hard people are willing to try, of how much of an effort they are planning to exert, in order to perform the behavior”* [2], p.181. Consumers again determine the type of product to buy because of ecological intentions such as the quest for healthier options, environmental consciousness and sustainability [59]. A change in consumer behavior is often determined by the market trend [75]. The consumer’s ability to weigh the quality, price, value and availability as competitive commodities, is as a result of personal marketing behavior [63]. To enhance environmental-friendly lifestyle, there is the need to create awareness and use of green products which will lead to a shift from conventional products to green products [5, 11]. It is reported that green products have strong influence on overall green purchase intention [38]. Outstandingly, the green purchase intention (GPI) of consumers’ ultimately impacts their green purchase behavior. As consumers’ have environmental concerns, they intend to buy GPs so as not to destroy the ecosystem but to act favorably and protect it for posterity. More importantly, consumers’ green product awareness, price, availability, value and quality could greatly influence their GPI [3, 38]. In brief, consumers’ who consider environmental issues are eager, prepared and likely to pay more for green products if they are conscious of its green credentials, superior value, high quality, functional attributes, environmental concerns and performance [9, 67]. And so, green product awareness should be created to get consumers’ into GPI and succeeding green consumption [63].

## Methods

### Research design

A quantitative research method was used in this paper so as to test the relationships among the variables of importance [11]. The quantitative research method is suitable in a scenario where the study involves more subjects. And this makes results generalization possible, while using recognized standards allows for replication of the research [41, 42].

### Procedures

In this study, the target population comprised students of the five departments of a University’s Faculty of Business and Management Studies. These five departments were: accountancy, marketing, secretaryship and management studies, communication studies, and procurement and supply chain management. The students of these five departments resided in the university’s halls of residence, the suburbs of the Municipality and other towns near the university. The ages of these students ranged from 18 to 31 plus. The ages of the undergraduate students ranged from 26 to 31 + because, in the university where the study was conducted and as in all universities in Ghana, there are students who are matured, older and advanced in age. These students are already holding Diploma and Higher National Diploma certificates and are already working in various organizations. As a result, the university gives them the opportunity in the evenings and weekends to do a two year top-up leading to bachelor’s degree. Again, the university has a policy of giving admission to students aged 25 + who are classified as “matured students” to study for a four-year Bachelor degrees. The study was conducted from October 15, 2019, to January 27, 2020. Data were collected by the use of an online survey.

The study targeted the entire population of students (1,109) at the Faculty consisting of five departments. The study attempted to use all the students for the research. However, after engaging/contacting them there were 550 who were ready and willing to be part of the study. Out of the 550 who were given the questionnaire via email, 478 returned their questionnaire and, thus, used in this study. This gave a response rate of 86.9%. This response rate is quite in line with past email-based research reply rates [20, 49]. The structured questionnaires were divided into sections, A and B. The section A was on statements related to the six variables: green product awareness (GPAW), green perceived price (GPP), green perceived availability (GPA), green perceived value (GPV), green perceived quality (GPQ) and green purchase intention (GPI), while section B was about demographics. All the closed-ended questions consisted of 30 items. Illustrated in Table 1 are the respondents’ demographics.

**Table 1:** Respondents’ demographics (*N* = 478)

Variable	Frequency	Percentage
Age		
18–21	181	37.9
22–25	253	52.9
26–30	32	6.7
31 +	12	2.5
Gender		
Male	220	46.0
Female	258	54.0
Education		
Diploma	142	29.7
Higher national diploma	202	42.3
Bachelor degree	134	28.0

These questionnaires were developed using the Google forms platform which were later distributed via emails to the participants. It is worth mentioning that the questions were simple, carefully modified and concisely worded to avoid ambiguity and formatted to avoid errors [23]. Moreover, other procedural approaches such as confidentiality, anonymity and the indication that there were no right or wrong answers written as part of the purpose and instructions to respondents were used to mitigate common method bias (CMB) [60]. Finally, inner variance inflation factor (VIF) was the statistical technique used to remove CMB. The predictor variables’ VIF ranged from 1.703 – 3.002, and this fell within the acceptable range [32].

### Measures

The thirty (30) Likert-type measurement items that were used in this research were adopted and modified from previous studies. This was done in order to improve the study’s quality, validity and reliability. With regard to all the variables, GPAW, GPP, GPA, GPV, GPQ and GPI, the questionnaires were adapted from studies such as [3, 68, 81]. It is notable that a 5-point Likert-type scale that ranged from 1 to 5, with 1 being “Strongly Disagree” to 5 being “Strongly Agree” was used to measure the entire constructs and five (5) items were equally used to measure each variable.

### Data analysis

The analysis of the data collected was done by using SPSS version 23.0 and structural equation modeling (SEM) using SmartPLS 3.0. Anderson and Gerbing [6] proposed two-phase process of SEM, namely the measurement model and the structural model. Consequently, using Smart PLS-SEM in this study looks at the aforesaid two levels of analysis. The measurement model links the observed variables to their identifiable latent variables, whereas the structural model joins the latent variable such as GPI to other latent variables such as GPAW, GPA, GPV, GPQ and GPP. The number of participants needed before SEM can be used remains debatable. Most scholars have suggested that a sample size of 200 participants is necessary for SEM to be used [37, 79]. Therefore, the sample size in this study (478) meets the criteria for SEM’s use [14]. To test the suitability of the model constructs with the items, the internal consistency, convergent validity and discriminant validity were examined.

## Results and discussion

**Fig. 1:**
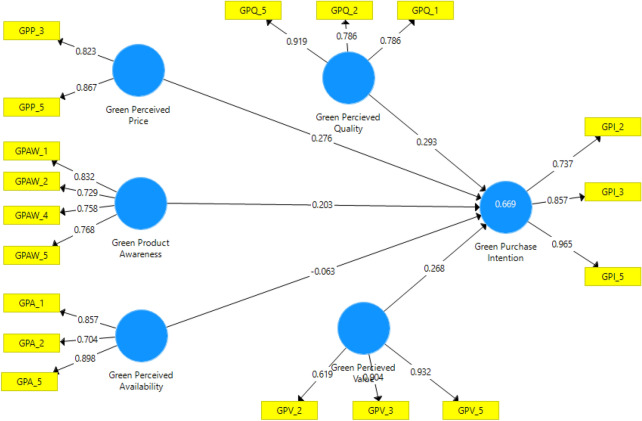
Tested research model

The SmartPLS estimates were used to test the hypotheses through the measuring of the path, strength and the significance level of the path coefficient. Figure 1 illustrates the test of the research model.

### Respondents’ demographics

The ages of the respondents were from 18 to 21 years (181) which represents 37.9%, 22–25 years (253) which is 52.9%, 26–30 years (32) with 6.7% and over 31 years (12) with 2.5%. There were 220 (46%) males and females 258 (54%) students from different departments. With regard to education level, 142 (29.7%) were diploma students, 202 (42.3%) were higher national diploma students, whereas 134 (28%) were bachelor degree students. Table 1 shows the respondents demographics.

### Measurement model

The SmartPLS-SEM is very robust, hence, its use in this study in order to better justify the hypotheses with deeper testing, integrity and contribution to green product awareness and green consumerism literature being studied [65]. To get reliable and dependable results from the SEM, it is vital to assess the constructs reliability and validity. The convergent validity and the discriminant validity checks were done to evaluate the constructs validity.

#### Reliability analysis

Having carefully scrutinized the outer loadings, the following items: two factors of green perceived availability (GPA_3 = 0.506, GPA_4 = 0.520), three factors of green perceived price (GPP_1 = 0.526, GPP_2 = 0.506, GPP_4 = 0.563), one factor of green product awareness (GPAW_3 = 0.542), two factors of green perceived value (GPV_1 = 0.547, GPV_4 = 0.565), two factors of green perceived quality (GPQ_3 = 0.343, GPQ_4 = 0.560) and two factors of green purchase intention (GPI_1 = 0.573, GPI_4 = 0.568) were deleted because they had low loadings which fell short of the threshold (< 0.60) as recommended by [28]. When the abovementioned low outer loadings were removed, the composite reliability (CR) coefficients of the constructs (green perceived availability, green perceived price, green product awareness, green perceived value, green perceived quality and green purchase intention) ranged from 0.833 to 0.892, and these CR coefficients indeed meet the suggestion made by [28].

#### Convergent validity

The SmartPLS algorithm was used to compute the outer loadings composite reliability (CR) and the average variance extracted (AVE). Thus, using the AVE, CR and outer loadings, the convergent validity was measured. The results showed that the AVE stretched from 0.597 to 0.736 and this meets the criterion for the convergent validity. As shown in Table 2, the outer loadings, CR and AVE values satisfied [28] proposals, (< 0.60) as factor loadings, (> 0.50) for AVE, while CR is (> 0.70).

**Table 2:** Outer loadings, reliability analysis and AVE

Variables	Items	Outer loadings	CR	AVE
Green perceived availability (GPA)			0.863	0.679
	GPA_1	0.857		
	GPA_2	0.704		
	GPA_5	0.898		
Green products awareness (GPAW)			0.855	0.597
	GPAW_1	0.832		
	GPAW_2	0.729		
	GPAW_4	0.758		
	GPAW_5	0.768		
Green perceived price (GPP)			0.833	0.714
	GPP_3		0.823	
	GPP_5		0.867	
Green perceived quality (GPQ)			0.871	0.694
	GPQ_1	0.786		
	GPQ_2	0.786		
	GPQ_5	0.919		
Green perceived value (GPV)			0.866	0.690
	GPV_2	0.619		
	GPV_3	0.904		
	GPV_5	0.932		
Green purchase intention (GPI)			0.892	0.736
	GPI_2		0.737	
	GPI_3		0.857	
	GPI_5		0.965	

#### Discriminant validity

In the measuring of the discriminant validity, the Fornell–Larcker benchmark of comparing square root of AVE with the constructs’ correlations was used. The AVE square roots of the entire constructs were beyond the squared correlations among the constructs, and this indicates satisfactory discriminant validity [28]. This is because all the items loaded strongly on their own than others. Table 3 displays the highlighted AVE square roots in a diagonal way.

**Table 3:** Fornell–Larcker criterion

Constructs	GPA	GPP	GPQ	GPV	GPAW	GPI
GPA	**0.824**					
GPP	0.812	**0.845**				
GPQ	0.518	0.624	**0.833**			
GPV	0.548	0.668	0.615	**0.831**		
GPAW	0.591	0.592	0.435	0.518	**0.773**	
GPI	0.580	0.707	0.686	0.703	0.595	**0.858**

*NB* diagonal values which have been highlighted are square root of AVE, while the rest are inter-construct correlations

### Structural model

The second aspect of PLS-SEM was to build and assess the structural model (SM). In brief, it is the supposed causation between the dependent construct and the independent constructs and this SM has been used to test the hypotheses in this study. To assess the SM, the algorithms and bootstraps were re-calculated and this occurred after some of the items/indicators have been deleted. The r-square, f-square, multicollinearity, standard deviations, the t-values, the p-values and the path coefficients were all completed, and these results are subsequently presented in Tables 4, 5, 6 and 7, respectively. The estimated t-statistics values and p-values are used to test the structural paths.

**Table 4:** R^2^ results

Endogenous variable	*R*^2^		Adjusted *R*^2^
GPI	0.669		0.666

**Table 5:** F^2^ (effect size) results

	*F*^2^	Effect size
GPA	0.004	Small
GPP	0.058	Small
GPQ	0.140	Small
GPV	0.102	Small
GPAW	0.073	Small

**Table 6:** Multicollinearity (inner VIF) results

	*VIF*
GPA	2.109
GPP	3.002
GPQ	1.856
GPV	2.117
GPAW	1.703

**Table 7:** Hypotheses testing summary

Hypothesis	Path coefficients	SD	T-Statistics	*P*-values	Results
H1: GPAW → GPI	0.203	0.041	4.913	0.000***	Supported
H2: GPP → GPI	0.276	0.065	4.239	0.000***	Supported
H3: GPA → GPI	− 0.063	0.047	1.345	0.179	Not supported
H4: GPV → GPI	0.268	0.050	5.375	0.000***	Supported
H5: GPQ → GPI	0.293	0.043	6.756	0.000***	Supported

^***^Significant at 0.001 level

#### R-square

**Fig. 2:**
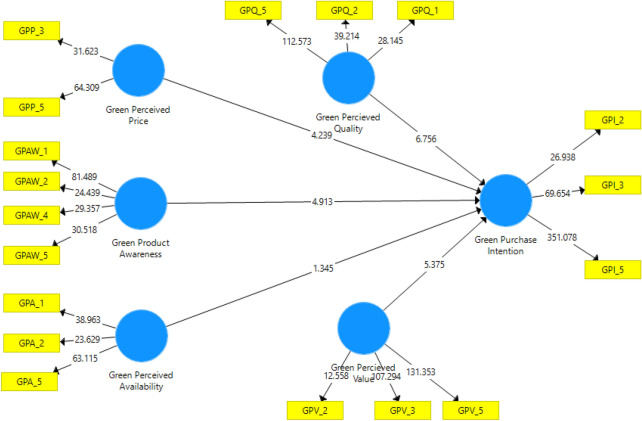
Bootstrapping (inner model and t-statistics)

R^2^ is the principal criterion used to evaluate the structural model [18]. The R^2^ revealed that the variance in the endogenous construct (green purchase intention – GPI) is explained by the five exogenous constructs (green perceived availability, green perceived price, green perceived quality, green perceived value and green product awareness). The R^2^ value of the endogenous variable, GPI (66.9%) in the current study is presented in Table 4, and it shows a very satisfactory level of prediction as suggested by [18] that a significant tolerable R^2^ should be greater than 0.26 (26%) for variance explained. This means that the descriptive power for GPI is acceptable among university students. The results derived from the SmartPLS bootstrapping, the structural model and its t-statistics are shown in Fig. 2.

#### F-square (effect size)

The effect size was measured using *F*^*2*^. With effect sizes, the values 0.00 – 0.15 mean small size, 0.15 – 0.35 mean medium and over 0.35 mean large effect [28, 65]. This study’s *F*^*2*^ values show that all the endogenous variables GPA (0.004), GPP (0.058), GPQ (0.140), GPV (0.102) and GPAW (0.073) which had small effect sizes.

#### Multicollinearity (inner VIF)

The predictor variables’ variance inflation factor (VIF) ranged between 1.703 and 3.002, and this fell within the acceptable range where VIF values less than 5.0 mean no multicollinearity problem as acclaimed by [28]. Therefore, in this study multicollinearity was not a concern.

#### Hypotheses testing and analysis

The results in Table 7 and Fig. 1 indicate that the path coefficient between green perceived quality and green purchase intentions has the most significant path connection (GPQ—**> **GPI, H5, β = 0.293; p < 0.001). Following closely is the path green perceived price to green purchase intention (H2: GPP—**> **GPI, β = 0.276; p < 0.001). The rest of the path relationships are as follows: green perceived value to green purchase intentions (H4: GPV—**> **GPI, 0.268; p < 0.001), green product awareness to green purchase intentions (H1: GPAW—**> **GPI, 0.203; p < 0.001) and, finally, green perceived availability to green purchase intentions (H3: GPA—**> **GPI, β = -0.063; p > 0.001). It is acknowledged that when the t-statistics is less than 1.96 and the p-value is greater than 0.05, then that particular hypothesis is not statistically significant. Looking at Table 7, it is notable that the t-value (1.345) of H3 fell below 1.96, while its p-value (0.179) is more than 0.05. Consequently, it is hereby concluded that H3 is not statistically significant and, therefore, H3 is not supported. Conversely, the results of H1, H2, H4 and H5 are all statistically significant because their t-values are more than 1.96 with their p-values also less than 0.05. As a result, hypotheses H1, H2, H4 and H5 are supported at 0.001 significant levels. Illustrated in Table 7 are the hypotheses results.

## Discussion

The results point out that 66.9% of the variance in the dependent variable (green purchase intention) is explained by the five predictors (green perceived availability, green product awareness, green perceived price, green perceived quality and green perceived value). This implies that this study explains the green purchase intention of university students in an emerging country far better [40]. While some studies have looked at the influencing factors that predict green purchase intentions [3, 9, 43, 52], the current study to the author’s knowledge is the first in an emerging economy, especially in a sub-Saharan African region, that examines the effect of green product awareness of university students’ on their green purchase intentions.

Results of this study indicate that green perceived quality (β = 0.293) has the utmost significant positive impact on university students’ green purchase intentions. In short, GPQ has the strongest effect on green purchase intentions. This means that students are eager to purchase green products when they identify that the quality is extraordinary if equated with conventional products. This result is in agreement with [8, 40, 50].

Besides, the second strongest predictor of university students’ green purchase intentions is green perceived price (β = 0.276). This finding shows that the relationship between GPP and GPI is positively significant. The university students’ keenness to buy green products is certainly triggered by their lucid evaluation of the price. Thus, to purchase green products, students will have to assess the price to see how beneficial it will be or whether it is value for money. This again indicates that when prices of green products are similar with conventional products, respondents will definitely have high GPI and for that matter switch to green products. However, low prices of green products will definitely command high occurrence of purchase and that students will switch to green products when the price is the same as their preferred brands. Therefore, price is one of the key features when it comes to the promotion of green products purchases and this confirms hypothesis 2. This result supports earlier study that has been identified by [4, 24].

In addition, there were positive path relationships between green perceived value to green purchase intentions (H4: GPV—**> **GPI, 0.268) and green product awareness to green purchase intentions (H1: GPAW**- > **GPI (β = 0.203). The paths of both had sturdier relationships which explained university students’ intention to purchase green products. Thus, students’ are anticipated to purchase products when the value is high, while students’ positive green perceived value of a product could cause higher increase in its purchase [3, 29, 30, 83]. Another implication is that green products awareness is a crucial element that influences and promotes university students’ green purchasing intentions. This finding is in line with prior research by [53, 70].

Conversely, the result showed that there was no statistically significant relationship between green perceived availability and green purchase intentions (H3: GPA—**> **GPI, β = -0.063, p-value = 0.179), whereas, at the same time, GPA was not positively linked to students’ green purchase intentions. The implication is that availability is not a critical influencing factor when it comes to university students’ green purchase intentions and subsequent behavior. In fact, this is very surprising, but what could have accounted for this? Although this result is supported by [44], yet, it is inconsistent with previous researches such as [4, 24, 40]. Consequently, it can be explained that in an emerging sub-Saharan African market like Ghana, traditional or conventional products are aplenty more than green products. It is, thus, suggested that businesses and governments in SSA embark on a great push to produce, provide and promote green products in order to safeguard the environment and to achieve the sustainable development goals 2030.

## Conclusions

The study examined the effect of green product awareness of university students’ on their green purchase intentions. The specific objectives of the paper were: (1) to identify whether awareness, price, availability, value and quality influence university students’ intention to purchase green products and (2) to investigate how awareness, price, availability, value and quality predict university students’ intention to purchase green products.

This study’s results suggested that green perceived quality has the most significant positive influence on university students green purchase intentions, whereas green perceived price is the second best predictor of university students green purchase intentions. However, the result revealed a negative relationship between perceived availability and green purchase intentions and that there was no statistically significant relationship between green perceived availability and green purchase intentions.

This research has made contributions to the extant literature on green consumerism by focusing on the green products awareness in emerging SSA markets such as Ghana. Again, as a contribution to knowledge, it is worth mentioning that the present moment of COVID-19 pandemic across the globe makes this study important as businesses and governments’ seek to encourage consumers/citizenry to purchase green products for environmental sustainability.

### Implications of findings

The study’s findings show that green product awareness effect on green purchase intention of university students is greatly driven by awareness, perceived price, perceived value, perceived quality and perceived availability. As regards the extent of the impact, it was found that GPQ and GPP correlated highly with GPI. Secondly, this study implies that it is among the few researches that consider how the awareness of green products by university students is impacted by GPQ and GPP in an emerging SSA market’s perspectives. Thus, the results show that GPQ significantly predicts GPI of university students and this indeed contributes to the literature.

The study’s findings revealed that GPQ and GPP have strong positive relationships with GPI of university students. As a result, policy makers, marketers, green manufacturers and governments could influence GPQ by launching marketing campaigns/promotions that will help to distribute knowledge on the benefits of green products. Those campaigns may improve consumers’ knowledge and understanding on green products and encourage positive approaches regarding its purchasing. It is necessary for consumers to be educated on the benefits that will accrue if they invest in green products. In so doing, they will be tempted to pay more for green products. Again, to resolve the issue of pricing, consumers’ could be offered discounts so as to motivate them to purchase green products.

### Limitations and future research

This study was conducted among undergraduate students, in a single university with the setting in one city, and therefore, the results cannot be generalized to all students. It is hoped that in future, more cities, more universities and all age categories of the population would be included. Also, this study did not stipulate particular kinds of green products, so, in future research, could cover specific green products. Finally, the research only considered students green purchasing intentions, so future research should focus on assessing real behavior of students who purchase green products frequently.

## Data Availability

The data sets used in this study are available upon request.

## A1: Questionnaires

### Green product awareness

GPAW_1: I have heard about green products.

GPAW_2: I have detailed knowledge and understanding about green products.

GPAW_3: I am aware of the difference between green products and conventional products.

GPAW_4: I buy green products instead of common/conventional products.

GPAW_5: I am aware that buying green products contributes to sustainable future.

## Green perceived price

GPP_1: I am more eager to pay more for green products.

GPP_2: I like green products but they are expensive.

GPP_3: Price is a major concern for me to go for green products.

GPP_4: Even though I like to buy green products but I cannot afford them.

GPP_5: I will switch to green products if it is available at the same price compared to my favorite brands.

## Green perceived availability

GPA_1: Green products are seen very often in my community.

GPA_2: Green products can easily be found in my community.

GPA_3: I have no difficulty in finding green products in my community.

GPA_4: I cannot find green products in places where I do shopping.

GPA_5: I may purchase green products only if they are simply available.

## Green perceived value

GPV_1: Green products give me extra value.

GPV_2: Green products have high value.

GPV_3: Green products give me more benefits than other products.

GPV_4: Green products environmental functions provide good value to me.

GPV_5: Green products have more environmental concern than non-green products.

## Green perceived quality

GPQ_1: Green product’s quality is superior to conventional ones.

GPQ_2: Green product’s quality is reliable compared to conventional ones.

GPQ_3: Green product’s quality is effective and stable compared to conventional ones.

GPQ_4: Green product’s quality is extraordinary compared to conventional ones.

GPQ_5: Green product’s quality is far better than conventional ones.

## Green purchase intention

GPI_1: I will likely purchase green products next month.

GPI_2: I intend to switch to a green variety of a product.

GPI_3: I am willing to purchase green products for personal use.

GPI_4: I will make an effort to purchase green products for my own use.

GPI_5: I plan to purchase green products for they do not pollute the environment.

## Abbreviations

GC: Green consumer
GPA: Green perceived availability
GPAW: Green product awareness
GP: Green product
GPI: Green purchase intention
GPP: Green perceived price
GPV: Green perceived value
GPQ: Green perceived quality
SDG: Sustainable development goal
SEM: Structural equation modeling
SSA: Sub-Saharan Africa
